# Measurement properties of the EQ-5D-3L, EQ-5D-5L, and SF-6Dv2 in patients with late-onset Pompe disease

**DOI:** 10.1007/s10198-024-01682-2

**Published:** 2024-03-12

**Authors:** Richard Huan Xu, Nan Luo, Dong Dong

**Affiliations:** 1 Department of Rehabilitation Sciences, Hong Kong Polytechnic University, Hong Kong SAR, China; 2 Saw Swee Hock School of Public Health, National University of Singapore, Singapore, Singapore; 3 JC School of Public Health and Primary Care, Faculty of Medicine, The Chinese University of Hong Kong, Hong Kong SAR, China

**Keywords:** EQ-5D, SF-6D, Measurement properties, Glycogen storage disease, Pompe disease

## Abstract

**Objective:**

The objective of this study was to evaluate the psychometric properties of the EQ-5D (3L and 5L) and SF-6Dv2 in a group of Chinese patients with late-on Pompe disease (PD), and compare their performance in this patient group.

**Methods:**

The data used in this study were obtained from a web-based and cross-sectional survey conducted in China. All participants completed the 3L, 5L, and SF-6Dv2. Information about their sociodemographic status and health conditions was also collected. The measurement properties were assessed by examining ceiling and floor effects, evaluating convergent validity, known-group validity, and test–retest reliability (Intraclass correlation coefficient [ICC] and Gwet’s AC).

**Results:**

A total of 117 PD patients completed the questionnaire. All dimensions of the 3L showed strong ceiling effects, ranging between 17.1 and 42.7%. All three measures showed good test–retest reliability, with ICC values ranging from 0.85 to 0.87. The Gwet’s AC values showed that four out of five dimensions of the 3L showed very good agreement. All hypothesized correlations between the 3L, 5L, SF-6Dv2, and items of WHODAS were supported, indicating satisfactory convergent validity. The 5L showed stronger correlations (|*r*|= 0.53–0.84) with WHODAS than the other two measures. The outcomes of ANOVA indicated that the 5L had higher *F*-statistics than the 3L and SF-6Dv2, indicating a stronger discriminant ability to differentiate most condition groups.

**Conclusion:**

The 5L demonstrates lower ceiling and floor effects, higher discriminant ability, and better convergent validity than the SF-6Dv2 and 3L in patients with PD. In addition, the 5L may generate a larger utility gain compared to the other two instruments when conducting cost-effectiveness analysis.

## Introduction

Generic preference-based measures (PBMs), such as EQ-5D and SF-6D, are widely used to measure health related quality of life (HRQoL) in different patient groups worldwide [1, 2]. Unlike condition-specific PBMs, generic PBMs are intended for use across conditions and treatments and to provide consistency and comparability for economic evaluations [3]. However, evidence for their use in rare disease populations is limited. In 2020, NICE’s methods review for HRQoL highlighted that there is insufficient published literature to provide evidence about the performance of the EQ-5D and other generic PBMs in rare and ultra-rare diseases [4]. This can pose a problem when using EQ-5D [5, 6] or SF-6Dv2 [7] to support economic evaluations of treatments for rare diseases.

The EQ-5D has both a 3-level (3L) and a 5-level (5L) version [5, 6], while the SF-6D has an updated version (SF-6Dv2) [7]. All these versions can generate a summarized utility score for calculating QALYs and use in cost-utility analysis for informing resource allocation decision-making [8]. Studies comparing the measurement performance of various PBMs for different diseases have been widely reported [9–12]. For example, studies have shown that the 5L has improved sensitivity and discriminatory power compared to the 3L, and that ceiling effects are reduced [13, 14]. Other studies have indicated that there is a significant difference between EQ-5D and SF-6Dv2, including that the descriptive system of SF-6D is more accurate than EQ-5D in specific patient groups [15]. The discriminatory power of 5L and SF-6Dv2 in different health indicators varies [16, 17].

Pompe disease (PD) is a rare genetic metabolic myopathy also known as glycogen storage disease type II, with an estimated incidence of 1 in 40,000 births [18]. PD results from a deficiency of an enzyme called acid alpha-glucosidase, which breaks down complex sugars in the body. This buildup occurs in organs and tissues, especially in muscles, causing them to break down. PD has two types: early-onset PD, which appears within a few months of birth, and late-onset PD, which appears later in a child’s life, or even into the teen years or adulthood. Those with early-onset PD rarely live past the age of 18 months, while patients with late-onset PD may survive up to the age of 30 years. If it starts in adulthood, they can live up to 50 years of age. Patients with PD usually have skeletal and/or respiratory muscle weakness, as well as symptoms such as pain and fatigue. Most eventually become wheelchair-bound or require respiratory support [18]. The resulting impact of PD on their HRQoL is substantial.

Assessing HRQoL can provide insight into identifying health needs, improving quality of care, understanding of medical needs, and monitoring of progress from a patient-centred perspective in rare disease care [19, 20]. However, few studies have evaluated the impact of symptoms and side effects on the HRQoL of patients with PD. An international survey of PD patients found that participants from the United Kingdom had significantly lower vitality scores but much higher scores in perceived general health than patients from America, Australia, Germany, and the Netherlands [21, 22]. Chen et al. indicated that adult Chinese patients with late-on PD had a lower physical HRQoL compared to their counterparts with other rare diseases [23]. Given the international and intercultural variations, it is important to investigate the HRQoL of PD patients in specific cultures and social environments of interest.

Evidence about the psychometric properties of the 3L, 5L, and SF-6Dv2 in patients with rare diseases, including PD, is scarce. A study conducted in the UK showed that 3L and SF-6Dv1 are suitable instruments for use in patients with PD, but they are not considered excellent [15]. However, there are no studies regarding the use of these PBMs in Asian populations. The lack of evidence hinders the use of these PBMs to generate the evidence needed by policy makers, researchers, and professionals for informed resource allocation. Therefore, this study primarily aimed to evaluate the psychometric properties of the 3L, 5L, and SF-6Dv2 in a group of patients with PD in China. The secondary aim was to compare the performance of these three measures in this patient group. With the revision of China’s orphan drug development practice [24], the importance of using a valid measure to examine HRQoL in this population is increasingly recognized by researchers and clinical professionals.

## Methods

### Data and participants

This study utilized data obtained from a web-based cross-sectional survey conducted in China between January and April 2023. The research team collaborated with the China PD Care Center (PDCC), the largest national patient organization for PD, to recruit participants from its internal network. All participants were required to be registered members of the PDCC and meet the following inclusion criteria: (1) ≥ 16 years old; (2) no cognitive impairment; (3) capable of completing the questionnaire independently; and (4) diagnosed with late-onset PD; and (5) able to provide informed consent. Participants who met the inclusion criteria were invited to join an online survey group, where the research team provided an introduction to the study’s aims, process, and expected outcomes. A survey link, including a consent form and questionnaire, was sent to all survey group members. Before beginning the survey, participants were required to read and agree to the informed consent statement, which was presented on the first page of the questionnaire. Information about participants’ background characteristics, HRQoL, and symptoms was collected. To assess test–retest reliability, all participants were invited to complete the EQ-5D and SF-6Dv2 1 week after the initial survey.

The research team has collaborated with PDCC to control the quality of the data. A research assistant checked the completion time (excluding those who completed the survey in less than 1/3 of the median survey length) and response pattern (avoiding flatlining, which is selecting the same answer for all questions) of each participant. Only the data that met the criteria were included for analysis. Reminders were sent to participants via online social platforms every 3 days until the survey was completed. Research team did not have access to the personal identity of participants, as only PDCC’s staff had access to such information. The study proposal and informed consent were approved by the Institutional Review Board of the Hong Kong Polytechnic University (Ref ID: HSEARS20220829001).

### Measures

#### Background information and clinical data

The participants’ demographic data were collected, including their sex, age, residence registry, marital status, number of children, educational attainment, and employment status. In addition, information about PD-related health conditions was obtained, such as the use of a wheelchair or ventilator, disability, difficulty standing up from a seated position, unstable walking prone to falling, difficulty changing from a lying position to a sitting position, difficulty lifting objects above the head, difficulty breathing, atrophy of the paraspinal muscles, and scapula alata. The list of symptoms and conditions was developed based on a literature review conducted by the research team, as well as discussions with PD patients, their caregivers, and managers of PDCC.

#### EQ-5D

The EQ-5D was used in this study to assess HRQoL [5, 6, 25]. All participants were requested to complete both 3L and 5L version. Their descriptive systems evaluate HRQoL based on five dimensions: mobility, self-care, usual activities, pain/discomfort, and anxiety/depression. The 3L and 5L versions provide three— (no problem, some problems, confined to bed/extreme problems/unable) and five— (no problems, slight problems, moderate problems, severe problems, and extreme problems/unable) response-level options, respectively. All health states can be converted into a summarised utility score between 0 (death) and 1 (full health) to facilitate cost-utility analysis. To calculate the utility score, the EuroQol suggested Chinese value sets were used for estimating the utility score for the 3L [26] and 5L [27], respectively. The range of utility scores for 3L and 5L is −0.149 to 1 and −0.391 to 1, respectively. We also administered the visual analogue scale (EQ VAS) to describe individual’s overall health status (0 [the worst health you can imagine]–100 [the best health you can imagine]).

#### SF-6Dv2

The SF-6Dv2 is a generic PBM consisting of six dimensions (physical functioning, role limitations, social functioning, pain, mental health and vitality) [7]. Each dimension can be rated using a five-response-level option, except for the pain dimension, which uses a six-response-level option. The Chinese utility value of the SF-6Dv2 (range: −0.535 to 1) was used to calculate the utility score in this study [28].

#### 12-Item World Health Organization Disability Assessment Schedule (WHODAS) 2.0

WHODAS 2.0 is a generic instrument that captures information about health and disability-related functioning in six life domains, including cognition (Item 3 and 6), mobility (Item 1 and 7), self-care (Item 8 and 9), social (Item 10 and 11), household (Item 2 and 12), and society (Item 4 and 5). In this study, the 12-item version of the WHODAS (WHODAS-12) was used. We employed the WHO simple scoring method that gives a 12-item WHODAS 2.0 score range from 12 to 60, where higher scores indicate higher disability or loss of function [29]. The score for each domain was calculated by adding the scores of the corresponding items within that domain.

### Data analysis

The central tendency, measured using mean and median, and variance, measured using standard deviation and interquartile range, were reported for 3L, 5L, and SF-6Dv2. The ceiling and floor effects were defined as the proportions of respondents who endorsed the “no problems” response option (full health, i.e., ‘11111’ for 3L or 5L and ‘111111’ for Sf6Dv2) and “extreme problems” response option (worst health, i.e., ‘55555’ for 5L, ‘33333’ for 3L, and ‘555655’ for SF-6Dv2), respectively.

To assess convergent validity, we tested the relationship between the 3L and 5L/SF-6Dv2 and WHODAS using Spearman’s rank correlation. The strength of the correlation was interpreted as weak (≤ 0.29), moderate (0.3–0.49), strong (≥ 0.5) [30]. We hypothesized that there would be a moderate to strong association between scales measuring similar health concepts, specifically:

1. 3L, 5L, or SF-6Dv2 utility score and WHODAS-12 sum score;
2. EQ-5D mobility dimension and WHODAS-12 mobility domain;
3. EQ-5D self-care dimension and WHODAS-12 self-care domain;
4. EQ-5D usual activities dimension and WHODAS-12 household domain;
5. EQ-5D anxiety/depression dimension and WHODAS-12 cognition domain;
6. SF-6Dv2 dimension physical function and WHODAS-12 mobility domain;
7. SF-6Dv2 role limitation dimension and WHODAS-12 self-care domains;
8. SF-6Dv2 social functioning dimension and WHODAS-12 social domain;
9. SF-6Dv2 mental health dimension and WHODAS-12 cognition domain.

Furthermore, we assessed the associations of dimensions between EQ-5D and SF-6Dv2. We assumed that there would be a moderate to strong association of dimensions that assess similar health concepts between EQ-5D, including 3L and 5L, and SF-6Dv2. Specifically:

10) EQ-5D mobility and SF-6Dv2 physical functioning;
11) EQ-5D self-care and SF-6Dv2 role limitation;
12) EQ-5D usual activities and SF-6Dv2 social functioning;
13) EQ-5D pain/discomfort and SF-6Dv2 pain;
14) EQ-5D anxiety/depression and SF-6Dv2 mental health.

We assessed the known-group validity of our study by examining the levels of both dimension and utility scores across different subgroups of symptoms and disability. We hypothesized that patients with symptoms or disabilities would report a higher proportion of problems and lower utility scores than those without. We used a Pearson’s Chi-squared test to determine whether the difference in proportion was statistically significant. In addition, we used the *F-statistic* derived from the ANOVA test to evaluate the relative efficiency of the EQ-5D and SF-6Dv2 utility scores. A higher *F-value* indicates higher discriminatory power.

Test–retest reliability of 3L, 5L, and SF-6Dv2 was assessed using Gwet’s agreement coefficient (Gwet’s AC) for dimension and intraclass correlation coefficient (ICC) for utility score [31]. The ICC model used was “Two-way mixed effects”, with a “Single measurement” type and an “Absolute agreement” definition. A coefficient less than 0.2 for Gwet’s AC is interpreted as poor agreement, 0.21–0.4 as fair, 0.41–0.6 as moderate, 0.61–0.8 as good, and greater than 0.8 as very good [32]. A value greater than or equal to 0.7 for ICC is considered to indicate good reliability [33].

A Bland–Altman (B–A) plot was also used to graphically describe the agreement between 3L, 5L, and SF-6Dv2 utility scores. All statistical analyses were performed using R (R Foundation, Austria), and a difference was considered statistically significant if the *P* value was less than 0.05.

## Results

### Participants’ characteristics

A total of 117 PD patients completed the questionnaire, resulting in a response rate of 87.3%. Approximately half of the participants were male, around 55% were over 30 years old, and 73% lived in rural areas. In addition, more than half (55.6%) had completed only secondary education or below, and nearly 77.8% were not actively employed (Table 1). The mean time for survey complete was approximately 15 min. No missing data were recorded because we have designed all the questions to be completed compulsorily.

**Table 1:** Participants’ background characteristics (*n* = 117)

	*n*	%
Sex		
Male	59	50.4
Female	58	49.6
Age		
16–20	20	17.1
21–30	33	28.2
31–40	46	39.3
> 40	18	15.4
Residence registry		
Urban	44	37.6
Rural	73	62.4
Marital status		
Single	69	59
Married	41	35
Widow(er)	7	6
Number of children		
0	74	63.2
1	30	25.6
2	13	11.1
Educational attainment		
Secondary or below	65	55.6
Tertiary or above	52	44.4
Employment status		
Active	26	22.2
Non-active	91	77.8

### Ceiling and floor effects

**Fig. 1:**
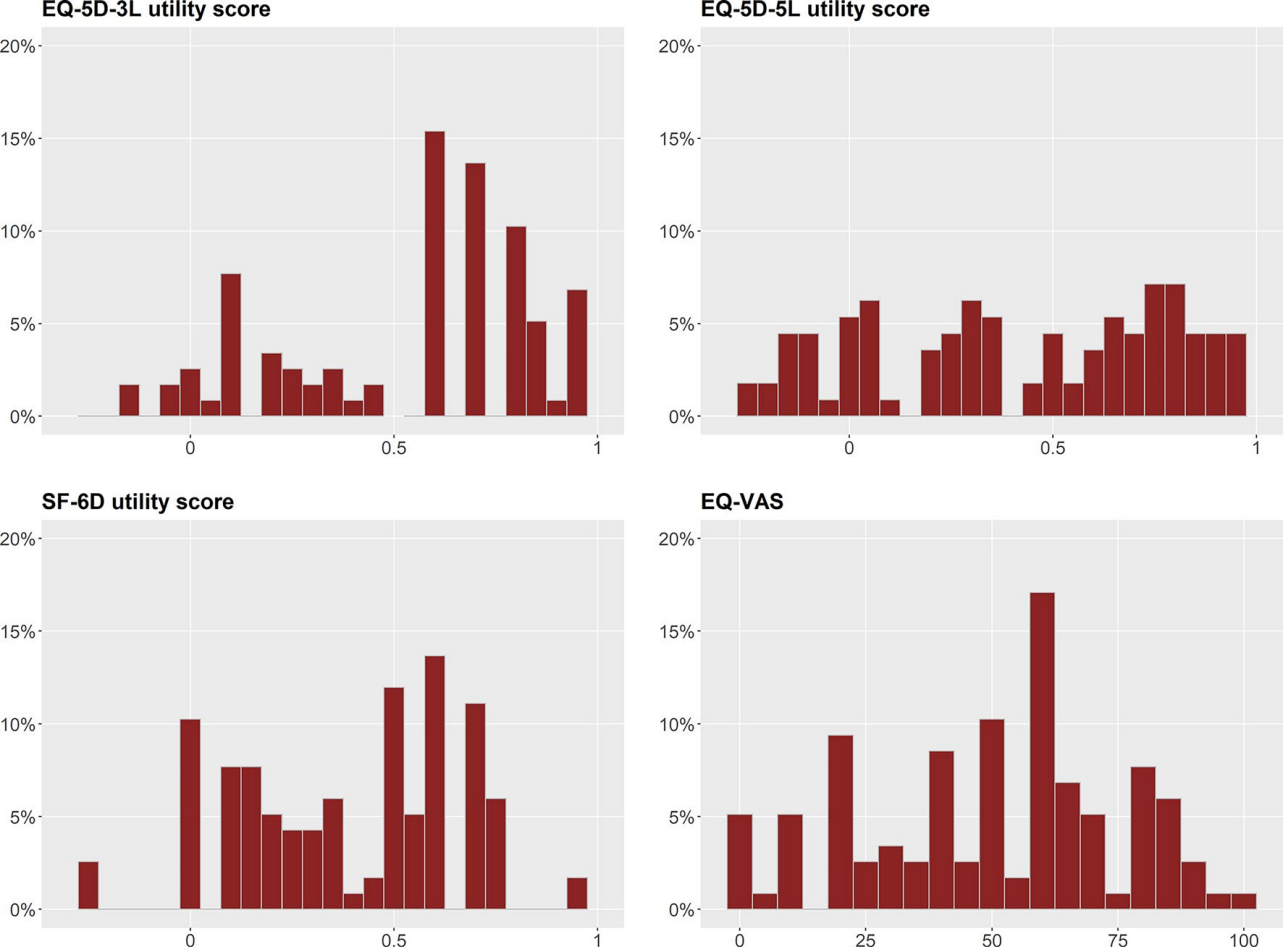
Distributions of utility score of 3L, 5L, and SF-6D and EQ VAS

The frequencies and percentages of reported problems for 3L, 5L, and SF-6Dv2 are presented in Table 2. Ceiling effects were identified for all dimensions of the 3L. Specifically, 42.7% reported no problems with self-care, followed by 30.8% indicating no problems with pain/discomfort. As for the 5L, self-care showed a moderate ceiling effect. No ceiling effects were observed for any dimension of the SF-6Dv2. Regarding floor effects, around 21.4% of participants reported unable to perform usual activity and confined to bed (“Mobility” dimension) for 3L, respectively. For 5L, participants reported the highest proportion of unable to wash or dress myself (“Self-care”) and unable to walk about (“Mobility”). Regarding SF-6Dv2, approximately 36.8% and 35% of patients with PD reported extreme problems in their social and physical functioning, respectively. The distribution of responses for similar dimensions between 5L and SF-6Dv2 is comparable. For instance, there are no ceiling effects for mobility in 5L and physical functioning in SF-6Dv2, but significant floor effects were observed. In addition, for anxiety/ depression in 5L and mental health in SF-6Dv2, no ceiling or floor effects were observed for either dimension. In terms of overall health status, 3L (6.8%) showed a higher proportion of full health status than 5L (0.9%) and SF-6Dv2 (0%). No participant selected ‘very severe pain’ in SF-6Dv2, the worst health for the SF-6Dv2 was ‘555555’. None of the measures presented significant ceiling or floor effects. The distribution of utility score of 3L, 5L, and SF-6Dv2 and EQ VAS is presented in Fig. 1.

**Table 2:** EQ-5D and SF-6D dimension and utility score profile

EQ-5D-3L	*n*	EQ-5D-5L	*n*	SF-6Dv2	*n*
*Mobility*		*Mobility*		*Physical functioning*	
No problems	22(18.8%)	No problems	14(12.0%)	Limited in vigorous activities not at all	4(3.4%)
Slight problems	70 (59.8%)	Slight problems	25 (21.4%)	Limited in vigorous activities a little	16 (13.7%)
Confined to bed	25 (21.4%)	Moderate problems	20 (17.1%)	Limited in moderate activities a little	14 (12.0%)
		Severe problems	29 (24.8%)	Limited in moderate activities a lot	42 (35.9%)
		Unable	29 (24.8%)	Limited in bathing and dressing a lot	41 (35.0%)
*Self-care*		*Self-care*		*Role limitation* *(Accomplish less than you would like)*	
No problems	50 (42.7%)	No problems	35 (29.9%)	None of the time	3 (2.6%)
Slight problems	46 (39.3%)	Slight problems	30 (25.6%)	A little of the time	13 (11.1%)
Unable	21 (17.9%)	Moderate problems	15 (12.8%)	Some of the time	36 (30.8%)
		Severe problems	13 (11.1%)	Most of the time	27 (23.1%)
		Unable	24 (20.5%)	All of the time	38 (32.5%)
*Usual activities*		*Usual activities*		*Social functioning* *(Social activities are limited)*	
No problems	20 (17.1%)	No problems	14 (12.0%)	None of the time	3 (2.6%)
Slight problems	72 (61.5%)	Slight problems	27 (23.1%)	A little of the time	16 (13.7%)
Unable	25 (21.4%)	Moderate problems	24 (20.5%)	Some of the time	26 (22.2%)
		Severe problems	29 (24.8%)	Most of the time	29 (24.8%)
		Unable	23 (19.7%)	All of the time	43 (36.8%)
				*Pain*	
				No pain	17 (14.5%)
				Very mild pain	24 (20.5%)
				Mild Pain	39 (33.3%)
				Moderate pain	32 (27.4%)
				Severe pain	5 (4.3%)
				Very severe pain	0 (0%)
*Pain/discomfort*		*Pain/discomfort*		*Mental health (Depressed or very nervous)*	
No problems	36 (30.8%)	No problems	18 (15.4%)	None of the time	6 (5.1%)
Slight problems	75 (64.1%)	Slight problems	43 (36.8%)	A little of the time	19 (16.2%)
Extreme problems	6 (5.1%)	Moderate problems	37 (31.6%)	Some of the time	56 (47.9%)
		Severe problems	13 (11.1%)	Most of the time	30 (25.6%)
		Extreme problems	6 (5.1%)	All of the time	6 (5.1%)
*Anxiety/depression*		*Anxiety/depression*		*Vitality*	
No problems	32 (27.4%)	No problems	10 (8.5%)	Worn out none of the time	2(1.7%)
Slight problems	76 (65.0%)	Slight problems	42 (35.9%)	Worn out a little of the time	9 (7.7%)
Extreme problems	9 (7.7%)	Moderate problems	42 (35.9%)	Worn out some of the time	46 (39.3%)
		Severe problems	13 (11.1%)	Worn out most of the time	46 (39.3%)
		Extreme problems	10 (8.5%)	Worn out all of the time	14 (12.0%)
Full health (11111)	6.8%	Full health (11111)	0.9%	Full health (111111)	0%
Worst health (33333)	1.7%	Worst health (55555)	1.7%	Worst health (555655)	0%
Best health (EQ VAS = 100)	0.8%	–	–	–	–
Worst health (EQ VAS = 0)	4.3%	–	–	–	–

### Test–retest reliability of 3L, 5L, and SF-6Dv2

A total of 110 PD patients participated in the baseline survey and completed the follow-up questionnaire 1 week later (response rate = 94%). The analysis using Gwet’s AC showed that good agreement of both 3L and 5L dimensions, except for “Pain/Discomfort”, which had a moderate agreement. Specifically, for 3L, four out of five dimensions showed very good agreement. For 5L, Gwet’ AC values ranged from 0.56 to 0.65. As for the SF-6Dv2, three out of six dimensions showed good agreement, while the other three showed moderate agreement. In addition, the ICC analysis also confirmed good test–retest reliability for all three measures (Table 3).

**Table 3:** Test–retest reliability of the Eq-5D and SF-6D

Measures	Mean (sd) [Median, range]	Gwet’s AC/ ICC (95% C.I.)
EQ-5D-3L		
Mobility	–	0.83(0.69,0.87)
Self-care	–	0.82(0.67,0.86)
Usual activities	–	0.82(0.68,0.87)
Pain/discomfort	–	0.8(0.68,0.86)
Anxiety/depression	–	0.72(0.56,0.77)
EQ-5D-5L		
Mobility	–	0.63(0.46,0.68)
Self-care	–	0.65(0.48,0.69)
Usual activities	–	0.65(0.48,0.69)
Pain/discomfort	–	0.56(0.38,0.6)
Anxiety/depression	–	0.65(0.48,0.7)
SF-6Dv2		
Physical functioning	–	0.74(0.59,0.79)
Role limitation	–	0.51(0.31,0.53)
Social functioning	–	0.59 (0.43,0.65)
Pain	–	0.64(0.46,0.68)
Mental health	–	0.63(0.46,0.67)
Vitality	–	0.57(0.4,0.62)
EQ-5D-3L utility score	0.54(0.27) [0.59, − 0.15–0.96]	0.87(0.82,0.91)
EQ-5D-5L utility score	0.36(0.41) [0.35, − 0.39–1.0]	0.85(0.76,0.91)
SF-6Dv2 utility score	0.39(0.27) [0.46, − 0.24–0.96]	0.85(0.78,0.9)
EQ VAS	49.9(25.3) [51.0, 0–100]	0.71 (0.58,0.8)

### Graphical agreement between measures

**Fig. 2:**
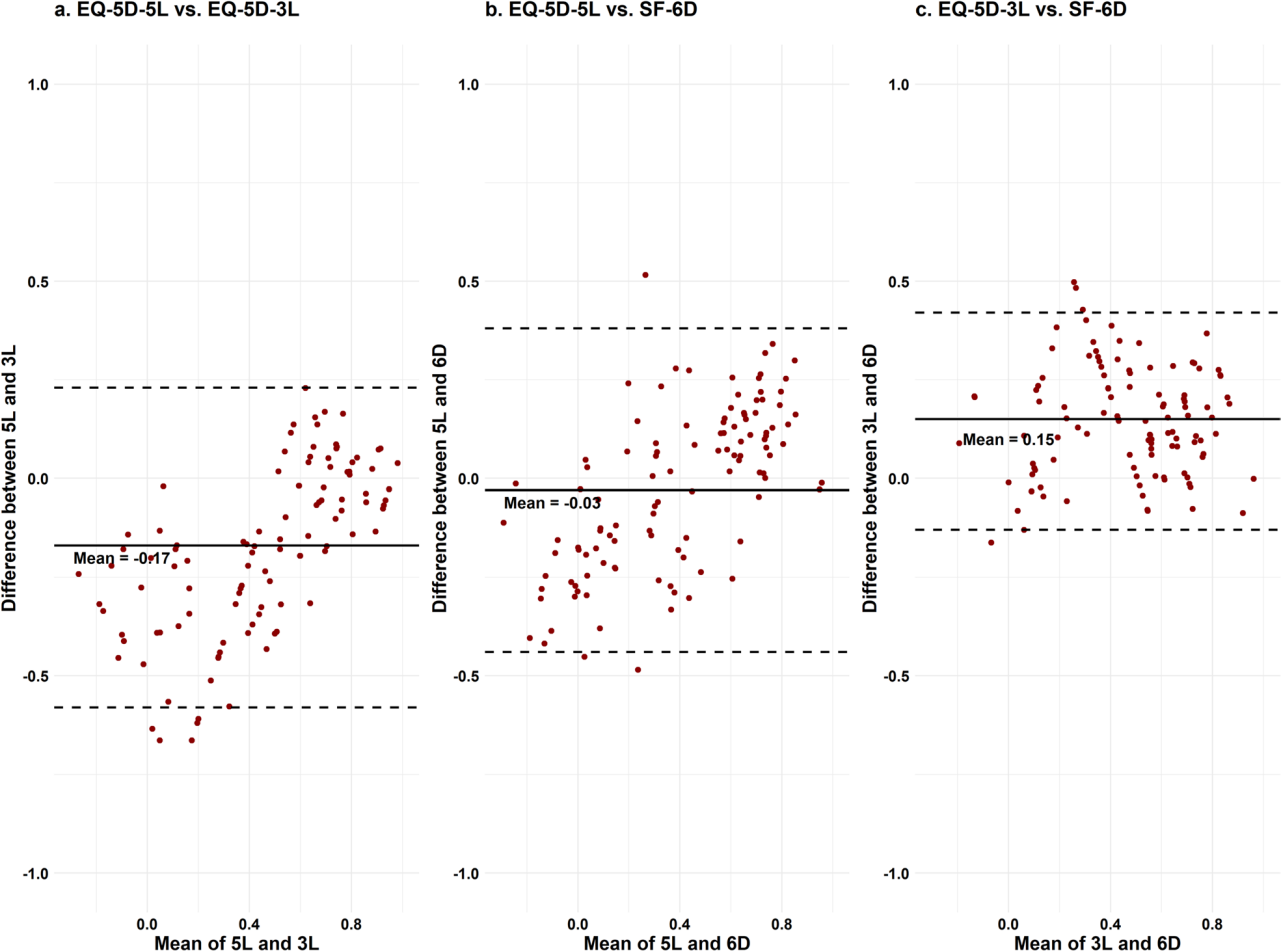
B-A plots for the agreement between measures

The acceptable agreement between three measures, graphically described by B-A plots, is presented in Fig. 2. There are nearly no observations outside the 95% limits of agreement, indicating an overall acceptable agreement. However, the agreement between 3 and 5L appears to be slightly weak at the lower end of the scale.

### Convergent validity of the measures

Table 4 presents the associations between 3L, 5L, and SF-6Dv2. There are very strong associations of dimension between 3 and 5L with the correlation coefficients ranged between 0.72 and 0.83. The dimensions of 3L and 5L showed strong correlation with SF-6D-related dimensions. In addition, the associations of EQ VAS with 3L, 5L, and SF-6Dv2 utility score were significant and strong.

**Table 4:** Correlations between EQ-5D and SF-6Dv2

Corelations	Values
EQ-5D-3L MO–EQ-5D-5L MO	0.79^***^
EQ-5D-3L SC–EQ-5D-5L SC	0.83^***^
EQ-5D-3L UA–EQ-5D-5L UA	0.75^***^
EQ-5D-3L PD–EQ-5D-5L PD	0.76^***^
EQ-5D-3L AD–EQ-5D-5L AD	0.72^***^
EQ-5D-3L MO–SF-6D PF	0.64^***^
EQ-5D-3L SC–SF-6D RL	0.65^***^
EQ-5D-3L UA–SF-6D SF	0.56^***^
EQ-5D-3L PD–SF-6D PA	0.73^***^
EQ-5D-3L AD–SF-6D MH	0.63^***^
EQ-5D-5L MO–SF-6D PF	0.67^***^
EQ-5D-5L SC–SF-6D RL	0.71^***^
EQ-5D-5L UA–SF-6D SF	0.64^***^
EQ-5D-5L PD–SF-6D PA	0.78^***^
EQ-5D-5L AD–SF-6D MH	0.77^***^
3L utility–5L utility	0.83^***^
3L utility–SF-6D utility	0.82^***^
5L utility–SF-6D utility	0.89^***^
3L utility–EQ VAS	0.42^***^
5L utility–EQ VAS	0.49^***^
SF-6D utility–EQ VAS	0.47^***^

^***^P < 0.001

The dimensions of 3L, 5L, and SF-6Dv2 were strongly correlated with WHODAS-12 domains. All hypothesized associations were addressed. The correlation coefficients for 3L, 5L, and SF-6Dv2 were in the range of 0.44 to 0.79, 0.53 to 0.89, and 0.45 to 0.72, respectively. The dimensions of 5L showed a stronger association with related-WHODAS-12 domains than 3L and SF-6Dv2. Moreover, the 5L utility score also showed a stronger correlation with the WHODAS sum score than the other two measures. The outcomes indicated good convergent validity for all three measures, but the 5L showed better convergent validity than the 3L and SF-6Dv2 (Table 5).

**Table 5:** Correlations between EQ-5D, SF-6D, and WHODAS-12

Hypothesized associations	EQ-5D-3L	EQ-5D-5L	EQ VAS	SF-6Dv2
MO–WHODAS mobility	0.61^***^	0.71^***^	–	–
SC–WHODAS self-care	0.79^***^	0.89^***^	–	–
UA–WHODAS household	0.50^***^	0.64^***^	–	–
AD–WHODAS cognition	0.44^***^	0.53^***^	–	–
PF–WHODAS mobility	–	–	–	0.50^***^
RL–WHODAS self-care	–	–	–	0.72^***^
SF–WHODAS social	–	–	–	0.58^***^
MH–WHODAS cognition	–	–	–	0.45^***^
WHODAS sum score	− 0.71^***^	− 0.84^***^	− 0.48^***^	− 0.78^***^

^***^P < 0.001

### Discriminatory power of the measures

Table 6 presents the discriminatory power of the utility score for the 3L, 5L, and SF-6Dv2 in known-group comparisons. The utility score for all measures exhibited a similar pattern of decrement as patients reported having clinical conditions compared to those who did not. The 5L demonstrated higher discriminant validity than the other two instruments across most symptom groups. However, the SF-6Dv2 was more effective in differentiating patients with two specific conditions—unstable walking and prone to falling, and difficulty breathing—than the EQ-5D. In terms of the magnitude of discriminant ability, the *F*-statistic confirmed that the 5L, with an *F*-value ranging between 5.8 and 54.4, had stronger discriminant ability than the 3L (*F*-value: 1.3–28.3) for all conditions, and the SF-6Dv2 (*F*-value: 4.4–35.8) for most conditions.

**Table 6:** Discriminatory power of the EQ-5D and SF-6D utility score

	*N*	EQ-5D-5L utility score (sd)	EQ-5D-3L utility score (sd)	SF-6Dv2 utility score (sd)
Wheelchair				
Using	41	0.06(0.29)	0.59(0.24)	0.22(0.2)
Used	17	0.37(0.38)	0.73(0.23)	0.37(0.26)
Never	59	0.56(0.36)	0.81(0.16)	0.51(0.25)
*F*-statistics		54.4	28.3	35.8
*P*-value		< 0.001	< 0.001	< 0.001
Ventilator				
Using	93	0.28(0.4)	0.68(0.24)	0.34(0.27)
Used	7	0.64(0.3)	0.87(0.11)	0.56(0.2)
Never	17	0.7(0.24)	0.85(0.07)	0.58(0.18)
*F*-statistics		16.5	10.7	15.8
*P* value		< 0.001	< 0.001	< 0.001
Disability				
Yes	63	0.17(0.36)	0.63(0.24)	0.28(0.24)
No	54	0.58(0.35)	0.83(0.15)	0.52(0.24)
*F*-statistics		37.8	27.5	27.6
*P* value		< 0.001	< 0.001	< 0.001
Difficulty standing up from a seated position				
No	37	0.68(0.32)	0.83(0.22)	0.57(0.22)
Yes	75	0.2(0.35)	0.67(0.21)	0.29(0.25)
*F*-statistics		12.3	4.3	10.6
*P* value		< 0.001	< 0.001	< 0.001
Unstable walking and prone to falling				
No	39	0.58(0.38)	0.79(0.24)	0.52(0.26)
Yes	73	0.24(0.37)	0.68(0.21)	0.31(0.25)
*F*-statistics		6.7	2.1	6.9
*P* value		0.01	0.15	0.009
Difficulty changing from lying position to sitting position				
No	35	0.56(0.33)	0.81(0.15)	0.52(0.24)
Yes	77	0.26(0.4)	0.68(0.24)	0.32(0.26)
*F*-statistics		5.8	3.1	4.8
*P* value		0.02	0.08	0.02
Difficulty in lifting objects above the head				
No	57	0.57(0.35)	0.8(0.18)	0.51(0.25)
Yes	55	0.14(0.35)	0.63(0.23)	0.25(0.23)
*F*-statistics		13	6.5	12.6
*P* value		< 0.001	0.01	< 0.001
Difficulty breathing				
No	36	0.59(0.33)	0.83(0.14)	0.55(0.21)
Yes	76	0.25(0.4)	0.67(0.24	0.31(0.27)
*F*-statistics		6.5	4.3	7.8
*P* value		0.01	0.04	0.005
Atrophy of the paraspinal muscles				
No	75	0.48(0.38)	0.76(0.22)	0.47(0.25)
Yes	37	0.1(0.34)	0.63(0.21)	0.22(0.24)
*F*-statistics		10	3.2	9.3
*P* value		0.002	0.07	0.003
Scapula alata				
No	67	0.46(0.39)	0.75(0.24)	0.46(0.25)
Yes	45	0.21(0.39)	0.67(0.2)	0.27(0.27)
*F*-statistics		6.7	1.3	4.4
*P* value		0.01	0.25	0.03

## Discussion

In this study, we found 5L demonstrates lower ceiling and floor effects, higher discriminant ability, and better convergent validity than the SF-6Dv2 and 3L for measuring HRQoL in patients with PD, a rare glycogen storage disease. This is the first study to head-to-head compare utility scores for the three most widely used PBMs in a patient population with a rare disease. These findings provide empirical evidence for selecting these generic PBMs to evaluate treatment and policies aiming at improving the HRQoL for patients with PD.

In the present study, no measures showed significant ceiling or floor effects. However, a lower proportion of patients reported full health status using 5L than using 3L, but higher than using SF-6Dv2. Compared to studies examining the measurement properties of the EQ-5D in other rare diseases, the ceiling and floor effects of 3L, 5L, and SF-6Dv2 in PD were much lower than in previous studies of Duchenne muscular dystrophy [34], haemophilia [35], and spinal and bulbar muscular atrophy [36].

Both EQ-5D and SF-6Dv2 showed a good convergent validity, and the improvement from 3 to 5L is significant in terms of the strength of association between EQ-5D dimensions and WHODAS item. This is partially consistent with previous findings that the improvement of convergent validity of 3L to 5L is significant but only to a limited extent [9, 37–40]. Currently, no PD-specific HRQoL instrument exists. Therefore, in this study, we selected the WHODAS, which is recommended in clinical practice guidelines for measuring functioning in patients with PD or other glycogen storage diseases [41, 42]. However, it is worth considering the inclusion of other instruments to assess the convergent validity of EQ-5D and SF-6Dv2 in this population. This is because some domains that affect HRQoL for patients with PD are missing in the WHODAS-12 (e.g., no dimension to measure pain).

In addition, 5L also demonstrated a stronger discriminant power than the 3L and SF-6Dv2, which is in line with previous findings in individuals with chronic low back pain [43]. Previous studies have confirmed that 5L has higher discriminant validity than the SF-6Dv1 estimated based on SF-12 in patients with haemophilia [35] or spinal and bulbar muscular atrophy [36]. Therefore, our study provides very first evidence on the relative validity or efficiency of the EQ-5D and SF-6Dv2 in patients with a relatively severe rare disease. In addition, another finding from the known-group validity analysis is that the mean difference between subgroups is consistently the largest with the 5L. For example, in patients using a wheelchair, the mean utility with the 5L is 0.06, while in those who have never used it, it is 0.56, resulting in a difference of 0.50. In comparison, the mean difference for the 3L is only 0.23, and for the SF-6Dv2, it is 0.29. This may suggest that in cost-effectiveness analyses, the utility gain is likely to be larger for the 5L than for the other two measures.

Our study demonstrated that both EQ-5D and SF-6Dv2 have good test–retest reliability. A previous study targeting cancer patients reported a stronger test–retest reliability of EQ-5D with a shorter time interval [37]. Both Gwet’s AC and ICC suggested that 3L is more reliable than 5L and SF-6Dv2, which is different from most of previous studies that 5L performed better than 3L or SF-6D [14, 44–46]. However, none of those studies were conducted in patients with a genetic rare disease. Our results may suggest that 5L is not more reliable than 3L for assessing patients with rare diseases. However, it is possible that the better test–retest result of 3L is false. If the health status of patients with rare disease was highly unstable, and the health status at the retest actually changed, the higher agreement between test and retest 3L scores would be due to the insensitivity of the scale. Hence, future investigators of test–retest assessments of HRQoL scales in patients with rare disease may consider using a shorter test–retest duration such as 2–3 days.

It is worth noting that in this study, there is insufficient evidence of the measurement properties of pain/discomfort as measured by the EQ-5D and pain as measured by the SF-6Dv2, since there are no corresponding pain or discomfort items in the WHODAS-12. Pain is a common symptom of PD, and in this study, most patients reported experiencing pain to some degree. Our findings are consistent with a previous study that indicated patients tend to inconsistently report their pain/discomfort using the EQ-5D [47]. In our study, the pain/discomfort dimension showed worse test–retest reliability than most other dimensions of the EQ-5D. However, for SF-6Dv2, the test–retest reliability of item “pain” is better than most other items, which may suggest that the discomfort part of the pain/discomfort dimension of the EQ-5D reduces the measurement properties of such instrument, as partially reported by various studies [47–49]. Given that patients with rare diseases are more likely to experience different types of pain or discomfort than the general population or patients with common diseases, and there are more than 100 different forms of discomfort identified, it is worthwhile to further assess the content validity of these concepts in this population.

Several limitations should be considered when interpreting the findings of this study. First, the sample was recruited from a volunteer pool via a patient organization’s internal network. These volunteers may be those patients in better health, which could have induced selection bias. Second, all questionnaires were self-reported by patients with PD, which may lead to recall bias. Third, although online surveys are commonly used in this type of research, the data quality may not be entirely guaranteed due to the web-based format. PD patients may not be fully engaged in a long survey due to poor physical and mental health, which could affect reliability of our findings. Last, considering we have collaborated with patient association to collect data, clinical information, such as comorbidities, were not collected based on their medical records. This may affect the validity of our findings.

## Conclusion

Overall, the 3L, 5L, and SF-6Dv2 instruments demonstrate satisfactory psychometric properties in Chinese patients with PD. The 5L showed better convergent and discriminant validity than the other instruments, while the 3L demonstrated better test–retest reliability than the 5L and SF-6Dv2. In addition, the 5L may generate a larger utility gain compared to the other two instruments when conducting cost-effectiveness analysis for interventions related to PD. Decision-makers should select an instrument based on the specific purpose of their research or practice.

## Data Availability

The datasets generated during and/or analyzed during the current study are available from the corresponding author on reasonable request.
